# Urinary Lithiasis Risk Assessment after Bariatric Surgery

**DOI:** 10.3390/jcm12124040

**Published:** 2023-06-14

**Authors:** Marie Tran, Khelifa Ait Said, Benjamin Menahem, Rémy Morello, Xavier Tillou

**Affiliations:** 1 Urology Department, CHU de Caen, Avenue de la Côte de Nacre, 14000 Caen, France; marietran75@gmail.com (M.T.); aitsaidkhelifa@gmail.com (K.A.S.); 2 Abdominal Surgery Department, CHU de Caen, Avenue de la Côte de Nacre, 14000 Caen, France; menahem-b@chu-caen.fr; 3 Biostatistic and Clinical Research Department, CHU de Caen, Avenue de la Côte de Nacre, 14000 Caen, France; morello-r@chu-caen.fr

**Keywords:** obesity, gastric bypass, urolithiasis, risk, screening strategies, questionnaire

## Abstract

Malabsorption is a consequence of gastric bypass (GB). GB increases the risk of kidney stone formation. This study aimed to evaluate the accuracy of a screening questionnaire for assessing the risk of lithiasis in this population. We performed a monocentric retrospective study to evaluate a screening questionnaire administered to patients who underwent gastric bypass surgery between 2014 and 2015. Patients were asked to answer a questionnaire that included 22 questions divided into four parts: medical history, episodes of renal colic before and after bypass surgery, and eating habits. A total of 143 patients were included in the study, and the mean age of the patients was 49.1 ± 10.8 years. The time between gastric bypass surgery and the completion of the questionnaire was 50.75 ± 4.95 months. The prevalence of kidney stones in the study population was 19.6%. We found that with a score of ≥6, the sensitivity and specificity were 92.9% and 76.5%, respectively. Positive and negative predictive values were 49.1% and 97.8%, respectively. The ROC curve showed an Area Under the Curve (AUC) of 0.932 ± 0.029 (*p* < 0.001). We developed a reliable and short questionnaire to identify patients at a high risk of kidney stones after gastric bypass. When the results of the questionnaire were equal to or greater than six, the patient was at a high risk of kidney stone formation. With a good predictive negative value, it could be used in daily practice to screen patients who have undergone gastric bypass and are at a high risk of renal lithiasis.

## 1. Introduction

Gastric bypass (GB) is a restrictive malabsorptive bariatric surgery. With more than 460,000 bariatric surgeries per year worldwide, it is booming due to the increase in global obesity [1]. A gastric bypass (Roux-en-Y gastric bypass) is a surgical procedure that creates a small pouch from the stomach and connects the newly created pouch directly to the small intestine to bypass the first portion of the small intestine to restrict food intake and reduce caloric absorption. Several studies have reported an increased risk of urinary stone formation after a GB. Urolithiasis is defined as the formation or presence of calculi in the urinary tract. The risk of a new stone event is multiplied by 2.13 after GB compared to the obese population (CI 1.30–3.49; *p* = 0.003) [2,3]. The largest cohort on this subject, with nearly 4636 American patients who underwent GB surgery, showed an increased risk of urolithiasis in operated patients, with 7.65% of them presenting with stones, whereas only 4.63% of obese non-operated patients did [4]. These results are consistent with those of a Canadian phone study showing a 7.3% incidence of renal colic after gastric bypass [5].

Urolithiasis after a gastric bypass is mainly explained by secondary enteric hyperoxaluria, which is defined by an increased secretion rate > 0.7 mmol/1.73 m^2^ per 24 h (measured in 24 h urine collection and adjusted to the oxalate excretion per 1.73 m2 of the body surface area) [2,6,7]. Other factors such as decreased hydration, oxalate and calcium consumption, vomiting, hypocitraturia, and hypocalciuria also play a role in urinary lithiasis. Currently, there is no screening protocol for the risk of urolithiasis after bypass surgery. Urolithiasis leads to increased medical morbidities and medical costs due to hospital stays and surgical procedures [2,4,5]. This study aimed to create and evaluate the accuracy of a simple and short retrospective questionnaire for urolithiasis risk assessment after bypass surgery. 

## 2. Materials and Methods

### 2.1. Population

We performed a monocentric retrospective study to investigate patients with obesity who underwent gastric bypass surgery at our institution between January 2014 and December 2015. Patients were adults operated on for gastric bypass after one year of follow-up with a psychologist, gastroenterologist, dietician, and sports educator. Before surgery, the patients were screened for other endocrinological disorders, including hypothyroidism, hyperparathyroidism, and diabetes. None of the patients had urinary calculi immediately before the gastric bypass. After surgery, the patients were followed-up at least every 6 months. A prospectively maintained database was filled in our department for all patients who underwent bariatric surgery. No systematic abdominal ultrasonography or CT scan were performed. We excluded patients who underwent other types of bariatric surgery, such as sleeve gastrectomy. Patients who underwent sleeve gastrectomy followed by a gastric bypass were included in this study. Patients with familial urinary lithiasis were excluded from this study. Patients were contacted by phone and asked to respond to the questionnaire. To ensure a long follow-up period, the interviews were conducted between January 2020 and January 2022. There were no exclusion criteria in this study. 

### 2.2. Questionnaire

**Figure 1:**
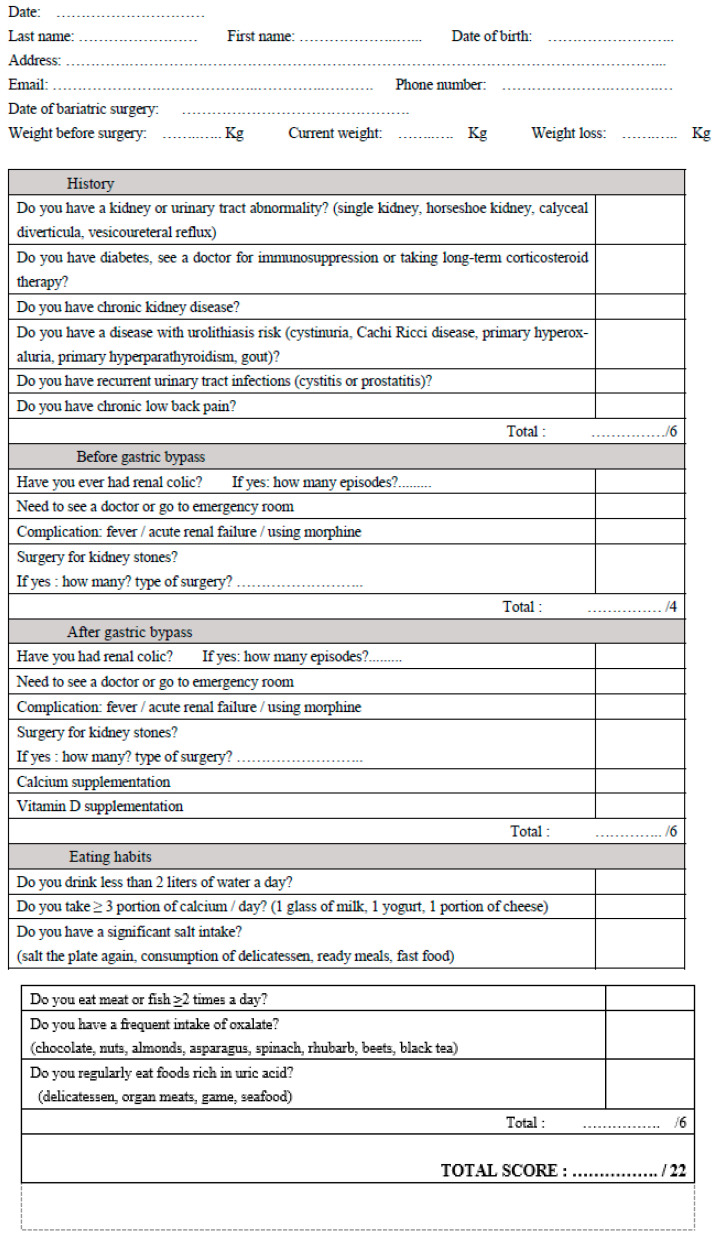
Evaluation questionnaire of urolithiasis risk after gastric bypass.

With no existing tools, we developed our own questionnaire (Figure 1). Two urologists developed a questionnaire based on the risk factors found in the scientific literature, recommendation guides, and our clinical experience. Before starting the study, 26 non-patients and non-medical professionals were asked to answer the questionnaire to correct the form and evaluate their understanding of each question by using a grid to check for appearance validity. Rewording was performed if necessary, which allowed us to complete the final questionnaire. The evaluation questionnaire included 4 parts, as shown in Figure 1. With no existing questionnaire weighing each item, it was decided for this first study that each question would count for one point. Given the questionnaire design, we were unable to perform any reliability tests. The questionnaire was translated into English for publication and has not yet been validated in English.

Data collection followed French law concerning retrospective non-interventional studies (Bioethics Law n° 2004-800 dated 6 August 2004, modified March 2012). The present study protocol was reviewed and approved by the Institutional Review Board of our University Hospital (no approval number was required for retrospective studies at the time of the study). Informed consent was obtained from all participants when they were enrolled (use of medical data).

### 2.3. Statistical Analysis

Data are presented as mean (±standard deviation (SD)) or as a percentage, depending on the quantitative or qualitative nature of the variables. The link between two qualitative variables was determined using Fisher’s exact test. Appearance validity was established using a grid of 26 non-patient non-medical professionals. Construct validity was assessed using principal component factor analysis (PCA) with varimax rotation on the four sub-scores to examine the factor structure of the questionnaire. The link between urolithiasis risk and the establishment of a threshold score for the questionnaire was based on a receiver operating characteristic (ROC) curve. The selected threshold corresponds to the point on the ROC curve that maximizes the sensitivity and specificity. The area under the curve (AUC) was presented as IC95. All diagnostic parameters were established based on this threshold. To consider the variability in the prevalence of lithiasis after GB, logistic regression analysis was performed to assess the risk of lithiasis (odds ratio [OR]) according to the threshold score of the questionnaire. A curve showing the evolution of positive and negative predictive values as a function of the variation in the prevalence of lithiasis risk was drawn. The IBM-SPSS software (version 23.0; IBM Corp., Armonk, NY, USA) was used to perform all analyses. All of the tests were performed bilaterally, and a value of *p* < 0.05 was considered statistically significant.

## 3. Results

Of the 304 patients who underwent surgery for gastric bypass between January 2014 and December 2015, 143 (47%) agreed to complete the questionnaire. There were 125 (87.4%) women and 18 (12.6%) men. Table 1 summarizes the characteristics of the study population. The mean age during the questionnaire was 49.1 ± 10.8 years. The mean time between gastric bypass surgery and completion of the questionnaire was 60.75 ± 4.95 months. The mean follow-up duration was 5.2 years. In total, 28 (19.5%) of the patients required treatment for urolithiasis after GB.

**Figure 2:**
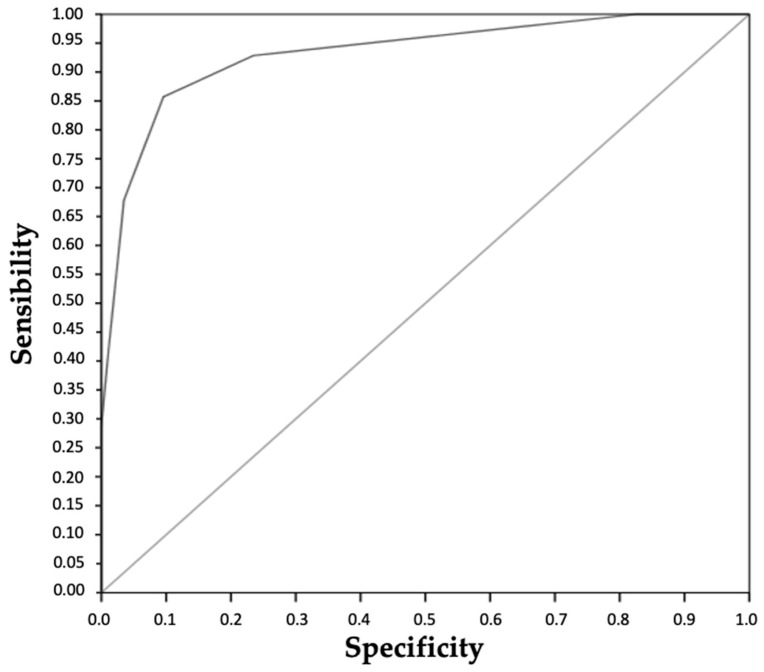
ROC curve of the renal stone risk.

A threshold of ≥6 in our screening questionnaire for 22 items provided the best sensitivity. The ROC curve (Figure 2) showed an area under the curve (AUC) of 0.932 ± 0.029 (*p* < 0.001) and an IC95 of [0.875–0.989]. In our questionnaire for screening for the risk of renal stones in obese patients who underwent surgery for gastric bypass, the significance threshold was 6. The prevalence of urolithiasis in patients with a questionnaire score of ≥6 was 19.6%. The sensitivity was 92.9%, with a positive predictive value of 49.1%. The specificity was 76.5%, and the negative predictive value was 97.8% (Table 2).

Table 3 shows the variables according to the questionnaire threshold of <6 or ≥6. The items highlighted by the established significance threshold were the presence of immunosuppression, repeated urinary tract infections, chronic lower back pain, episodes of renal colic before and after gastric bypass surgery, surgery required to treat kidney stones, vitamin D and calcium supplements, and excessive salt and oxalate intake (*p* < 0.005).

## 4. Discussion

Despite large studies [3,5], reviews [8,9,10], and meta-analyses [6,11], we did not find a tool to evaluate the risk of urolithiasis after GB. We established a rapid and reliable screening questionnaire to evaluate the risk of urolithiasis after gastric bypass. 

Immunosuppression (diabetes and long-term corticosteroid therapy) and repeated urinary tract infections (Questions 2 and 5) were the two significant risk factors listed in our questionnaire. Diabetes favors uric acid calculi, similar to obesity. Insulin resistance in type 2 diabetes (T2D) causes alterations in renal ammoniogenesis, and acid urine promotes the crystallization of uric acid stones. In total, 35.6% of patients with T2D have uric acid stones compared to 11.3% of patients without diabetes (<0.0001) [12]. Studies have shown that chronic urinary tract infections lead to the formation of stones composed of carbapatite and phosphate–ammoniac–magnesium [13]. Vitamin D and calcium supplements (Question 15), excessive salt (Question 19) and oxalate intake (Question 21) were other risk factors for urolithiasis in our study population. Calcium and vitamin D supplementation is common after GB. In theory, supplementation aims to prevent urinary stones by reducing intestinal absorption of oxalate. There was no significant difference in the risk of renal colic with or without vitamin D and calcium supplementation after GB due to poor compliance with supplementation treatment (approximately 50% in a Canadian study [5], a rate close to that in our study). Calcium intake can also be improved by following dietary advice, with sufficient food intake distributed throughout the day. The other methods of preventing kidney stones are as follows: sufficient water intake with drinking at least 2 L per day; correction of hypocitraturia with supplementation of 3 to 6 g per day of potassium citrate or a pharmaceutical supplement. Compliance with dietary advice is fundamental in preventing urolithiasis in this population [14].

Questions concerning urinary tract anomalies, chronic renal failure, and history of urolithiasis risk were not significant in our questionnaire. Few cases were identified, resulting in a lack of power in our statistical tests. This was a single-center study. The ideal investigation would be to carry out this questionnaire in a multicentric and prospective manner with systematic imaging before and after gastric bypass to search for all symptomatic and asymptomatic kidney stones. Recurrence of renal colic episodes is almost 50% at five years and requires surgical intervention in 20% of patients with urolithiasis [12]. Therefore, it would be interesting to carry out a study on a larger population, allowing screening for small kidney stones, preventing episodes of renal colic or acute obstructive pyelonephritis, and setting up uronephrological monitoring of people at risk of lithiasis. There is no consensus on urinary stone screening in patients with GBs [14]. It would be interesting in these patients to deepen the investigations with a biological assessment, such as 24 h urine or even renal ultrasonography with abdominal X-ray or non-contrast CT scan assessment. A monitoring protocol can be performed with urological or nephrological follow-ups associated with the usual dietary regimen. Indeed, stones formed after GB are of a particular type according to the Michel Daudon morpho-constitutional classification [15]. In the calcium oxalate monohydrate stone family (type I), there are five subfamilies (a, b, c, d, and e) with different origins and lithogenic mechanisms. The most frequent is type Ia oxalo-calcium calculus. They were dense calculi with 1200–1700 HU on CT. Thus, simple abdominal radiography may be useful for screening early urolithiasis.

With a high predictive negative value, our questionnaire is a good tool for selecting patients at no risk of urolithiasis. For patients with a score higher than 6, our study suggests that they are candidates for closer monitoring of urinary lithiasis to prevent or treat it at an early stage. One of the main limitations of this study was its retrospective design and declarative nature of the data, with a risk of information and selection bias. We could also add a validated quality of life questionnaire to this evaluation. In addition, the number of patients remains low, which affects the estimation of the value of the lithiasis OR for a score ≥ 6. A high AUC value and good sensitivity should be confirmed in future studies. Another limitation is the response rate of the study. With 47% of the patients agreeing to participate, this could constitute an inclusion bias. Moreover, with no pre-existing questionnaire in the field, we could not establish the number of participants needed to decrease bias influence. The present questionnaire needs to be validated on a larger scale first and then with different scores for each question. However, we believe that our results provide a simple and clinically relevant questionnaire for daily screening. This questionnaire remains to be evaluated within the framework of a validation study with multicenter and prospective inclusion of patients and systematic imaging before and after GB to meet the criteria of the American Educational Research Association [16]. 

## 5. Conclusions

Our study enabled us to develop a short and reliable questionnaire. Its simplicity provides good clinical relevance. It can be used in daily practice to screen patients who have undergone gastric bypass and are at a high risk of urolithiasis. When the results of the questionnaire were equal to or greater than six, the patient was at a high risk of kidney stone formation. Prevention of a large part of the complications of kidney stones, such as renal colic and acute obstructive pyelonephritis, which may destroy the kidney or cause chronic renal failure, can thus be ensured with early and specialized management. 

## Figures and Tables

**Table 1:** Patient demographics data.

Male Gender *n* (%)/Female	125 (87.4)/18 (12.6)
Mean age at time of study (years old) (±SD)	49.06 (±10.78)
Mean age at time of surgery (years old) (±SD)	44.28 (±10.76)
Mean time between surgery questionnaire (months)	60.75 (±4.95)
Mean weight before gastric bypass (kg)	122.22 (±19.35)
Mean current weight (kg)	82.78 (±19.09)
Mean weight loss (kg)	39.44 (±15.15)
Mean total score of questionnaire	5.34 (±2.17)

SD: Standard Deviation; Kg: Kilogramme.

**Table 2:** Cross table between questionnaire score and urolithiasis.

			Urolithiasis		Total
			No	Yes	
Questionnaire score	<6	Patients	88	2	90
		% Score	97.8%	2.2%	100%
		% Stone	76.5%	7.1%	62.9%
		Total (%)	61.5%	1.4%	62.9%
	≥6	Patients	27	26	53
		% Score	50.9%	49.1%	100%
		% Lithiasis	23.5%	92.9%	37.1%
		Total (%)	18.9%	18.2%	37.1%
Total		Patients	115	28	143
		Score	80.4%	19.6%	100%
		Lithiasis	100%	100%	100%
		Total	80.4%	19.6%	100%

Se = 92.9%; PPV = 49.1%; Sp = 76.5%; PNV = 97.8%; Se: sensibility; Sp: specificity; PPV: positive predictive value; PNV: predictive negative value.

**Table 3:** Variables according to the threshold of the screening questionnaire.

Variable		Score		*p*
		<6	≥6	
Gender	Female	80 (55.9)	45 (31.5)	0.603
	Male	10 (7.0)	8 (5.6)	
Urinary tract abnormality	no	90 (100)	53 (100)	-
	yes	0	0	
Immunosuppression	no	86 (60.1)	43 (30.1)	0.008
	yes	4 (2.8)	10 (7.0)	
Chronic renal failure	no	90 (62.9)	51 (35.7)	0.136
	yes	0	2 (1.4)	
Stone risk disease	no	89 (62.2)	50 (35.0)	0.144
	yes	1 (0.7)	3 (2.1)	
Recurrent urinary tract infection	no	81 (56.6)	38 (26.6)	0.006
	yes	9 (6.3)	15 (10.5)	
Chronic low back pain	no	69 (48.3)	18 (12.6)	0.001
	yes	21 (14.7)	35 (24.5)	
Renal colic before GB	no	89 (62.2)	35 (24.5)	0.001
	yes	1 (0.7)	18 (12.6)	
Stone surgery before GB	no	90 (62.9)	50 (35.0)	0.049
	yes	0	3 (2.1)	
Renal stone after GB	no	89 (62.2)	40 (28.0)	0.001
	yes	1 (0.7)	13 (9.1)	
Stone surgery after GB	no	90 (62.9)	49 (34.3)	0.018
	yes	0	4 (2.8)	
Calcium supplementation	no	62 (43.4)	15 (10.5)	0.001
	yes	28 (19.6)	38 (26.6)	
Vitamin D supplementation	no	30 (21.0)	6 (4.2)	0.001
	yes	60 (42.0)	47 (32.9)	
Insufficient hydratation	no	10 (7.0)	4 (2.8)	0.572
	yes	80 (55.9)	49 (34.3)	
Excess calcium intake	no	33 (23.2)	26 (18.3)	0.218
	yes	56 (39.4)	27 (19.0)	
Excess salt intake	no	79 (55.2)	31 (21.7)	0.001
	yes	11 (7.7)	22 (15.4)	
Excess protein intake	no	42 (29.4)	21 (14.7)	0.486
	yes	48 (33.6)	32 (22.4)	
Excess oxalate intake	no	61 (42.7)	22 (15.4)	0.003
	yes	29 (20.3)	31 (21.7)	
Excess uric acid intake	no	80 (55.9)	40 (28.0)	0.057
	yes	10 (7.0)	13 (9.1)	

N (%).

## Data Availability

Will be shared upon reasonable request to xavtillou@gmail.com.
